# Early detection of oral cancer: a key role for dentists?

**DOI:** 10.1007/s00432-022-03962-x

**Published:** 2022-03-06

**Authors:** Katrin Hertrampf, Martina Jürgensen, Stefanie Wahl, Eva Baumann, Hans-Jürgen Wenz, Jörg Wiltfang, Annika Waldmann

**Affiliations:** 1 Department of Oral and Maxillofacial Surgery, University Hospital of Schleswig-Holstein, Campus Kiel, Arnold-Heller-Str. 3, Building B, 24105 Kiel, Germany; 2 Institute of Social Medicine and Epidemiology, University of Lübeck, Ratzeburger Allee 160, 23562 Lübeck, Germany; 3 Department of Journalism and Communication Research, Hanover University of Music, Drama and Media, Expo Plaza 12, 30539 Hanover, Germany; 4 Department of Prosthodontics, Propaedeutics and Dental Materials, University Hospital of Schleswig-Holstein, Campus Kiel, Arnold-Heller-Str.3, Building B, 24105 Kiel, Germany

**Keywords:** Early detection, Oral cancer, Screening, Dentists, Feasibility

## Abstract

**Purpose:**

The majority of suspected malignant changes in the oral mucosa are detected by dentists in private practice. Statements regarding the effectiveness of visual examination of the oral cavity for early detection are not necessarily transferable between different health care systems. Our clinical-epidemiological and methodological aim was thus to conduct a prospective regional study in dental practices under everyday conditions, assess the frequency and type of oral mucosal changes, and evaluate the dental examination methodology.

**Methods:**

A prospective observational study was conducted, combining a feasibility study of early detection of oral cancer and its documentation with phase I ‘modelling’ to conceptualize complex interventions in health services research. Dentists in private practice continuously recruited patients over 6 months and used two different sheets for the documentation of suspicious lesions. Statistical analysis involved descriptive statistics and tests for differences (Welch test) or association (Chi-squared test).

**Results:**

Twenty-five dentists (mean age: 50 years, 24% females) participated in this study. Eleven dentists achieved the overall aim of recruiting 200 patients. Around 4200 patients (mean age: 52 years, 57.5% females) participated. The prevalence of suspicious lesions was 8.5%.

**Conclusion:**

It became apparent that a study in cooperation with dentists in private practice to generate clinical-epidemiological data on the early detection of oral mucosal lesions under everyday conditions can be carried out successfully. Further studies with a corresponding level of evidence should be carried out to be able to draw conclusions about the effectiveness of the early detection measure under everyday practice conditions.

## Introduction

The tumour of the oral cavity and pharyngeal region remains an underestimated public health problem. Worldwide, more than 529,000 affected individuals received this diagnosis (oral cavity including lips, pharyngeal region; ICD-10 C00-C14) in 2012, accounting for approximately 3.8% of all cancers (Shield et al. 2017). In Germany, the incidence of oral (including lips) and pharyngeal cancer has been on an upward trend in recent years, from about 10,000 previously to nearly 14,000 incidences (9720 men and 4180 women). In terms of the total number of all cancers, this tumour thus ranked seventh (3.8% of all malignancies) in men and 15th (1.8%) in women in the ranking of most common tumours in 2016. The five-year relative survival rate was only 47% in men and 63% in women (Robert Koch Institut 2019). One reason for this is that the majority of affected individuals do not consult a dentist, physician, or oral and maxillofacial surgeon until the tumour is at an advanced stage (Robert Koch Institut 2019). Diagnosis at an earlier tumour stage could not only improve the likelihood of survival, but also reduce therapy-related limitations in speaking, swallowing, and eating for patients (Cheung et al. 2021).

With regard to tumours of the oral cavity, the recommended visual clinical examination of the oral mucosa to establish an initial tentative diagnosis offers a form of early detection that is non-invasive, painless, and not time-consuming for the patient as well as having no side effects (Abadeh et al. 2019; Warnakulasuriya et al. 2015).

In Germany, this examination of the oral cavity is part of the routine semi-annual or annual dental examination required by the healthcare system. Patients who suspect a tumour in their oral cavity are often the first to contact their dentists in Germany. In both cases, i.e. whether arranging a routine check-up or a visit due to a suspected tumour, every insured person in Germany can actively make an appointment directly with a dentist. However, documentation of the inspection of the oral mucosa using a standardized form is not obligatory for dentists, as is the case for reporting dental status. Thus, there are no documentation sheets for the investigation of the oral mucosa available to dentists. Consequently, there is a lack of efficacy studies involving dentists in private practice and their patients, a situation not unique to Germany.

Although studies involving dentists in private practice have shown positive trends in early detection and underscored the importance of involving such dentists, it has not been possible to draw reliable conclusions on efficacy due to study design (Abadeh et al. 2019; Kerr et al. 2020; Warnakulasuriya et al. 2015). To date, there is only one study worldwide, from India, providing evidence on the efficacy of an oral mucosal screening measure (Sankaranarayanan et al. 2013). These results are very promising, but the study design is not transferable to health systems in other countries that do not use named ‘trained health workers’ for oral mucosal inspection instead of practicing dentists. Also, such results must always be discussed in the context of the respective healthcare system.

Thus, the study described here pursued both a clinical-epidemiological goal and a methodological one: the clinical-epidemiological goal of this prospective regional project was to generate regional epidemiological data regarding the frequency and type of oral mucosal changes via practicing dentists. The methodological objective was to test the feasibility of the study methodology focusing on the feasibility of the inspection of the oral mucosa and standardized recording of possible changes by practicing dentists. To this end, the practicability and acceptance of the study information and the documentation of possible oral mucosal changes as well as the manageability and comprehensibility of the reviewing documentation sheets had to be evaluated by dentists and patients.

## Material and methods

The study was conducted as a prospective observational study, and the study protocol was approved by the Ethical Committee of the Medical Faculty of the University of Kiel (AZ D468/15).

The study combined a feasibility study of early detection of oral cancer (inspection of oral mucosa and its documentation) with phase I ‘modelling’ (manuscript in preparation) to conceptualize complex interventions in health services research following the Medical Research Council (MRC) standard (Craig et al. 2008).

### Study population and recruitment

Dentists in private practice in Germany are members of so-called quality circles. These circles are independently established and organized by dentists and serve the purpose of regular collegial exchange. These circles vary greatly in terms of the number of participants, as there are neither upper nor lower limits on participation. Registration of a circle and its participants with the regional dental association is voluntary.

For recruitment of the dentists, six circles were randomly selected from registered quality circles in cooperation with the Schleswig–Holstein Dental Association. The initial approach to the head of the respective circle was made by the dental association and, in case of interest, a second contact was made by the project manager (KH). All heads contacted showed interest in the study and offered a personal meeting for further clarification.

Inclusion criteria were practice activity as a dentist or oral and maxillofacial surgeon within the federal state of Schleswig–Holstein (northern Germany), and written informed consent.

The inclusion criteria for the participation of patients from the respective practices was a presentation for a dental check-up during the documentation period and being of age at least 18 years old and member of statutory health insurance. In Germany, the six-monthly check-up forms part of the standard schedule of health insurance dental services and is, therefore, free of charge for patients. Patients who made an appointment in response to their own suspicion of oral mucosal changes were also included. Patients were only included once, even if they had more than one appointment during the study period. Privately insured patients were excluded from the study for three reasons. First, in Germany only about 9% are members of private health insurance. Second, the billing modalities of these two insurance systems are very different. Third, the data paths from the practices to the private health insurances are not yet sufficiently defined and standardised, and thus it is not possible to achieve sufficiently reliable data quality compared to statutory health insurances.

The first appointment was always documented. Written informed consent was required for all patients. Information regarding the study was provided and informed consent obtained by the participating dentists.

### Measures

At the beginning of the data collection, participating dentists were asked to provide sociodemographic information, including age, gender, previous duration of the dental practice, type of practice (single-dentist practice vs multiple practitioners), weekly working hours, and any additional qualifications.

Two differently designed documentation sheets were used to document the inspection of the oral mucosa. One was a purely text-based version, which had been developed in advance by two members of the working group (KH, AW). The other one was a graphic version which had been used in the Fifth German Oral Health Study (DMS V) (KH member of the expert group, permission to use was available).

**Fig. 1:**
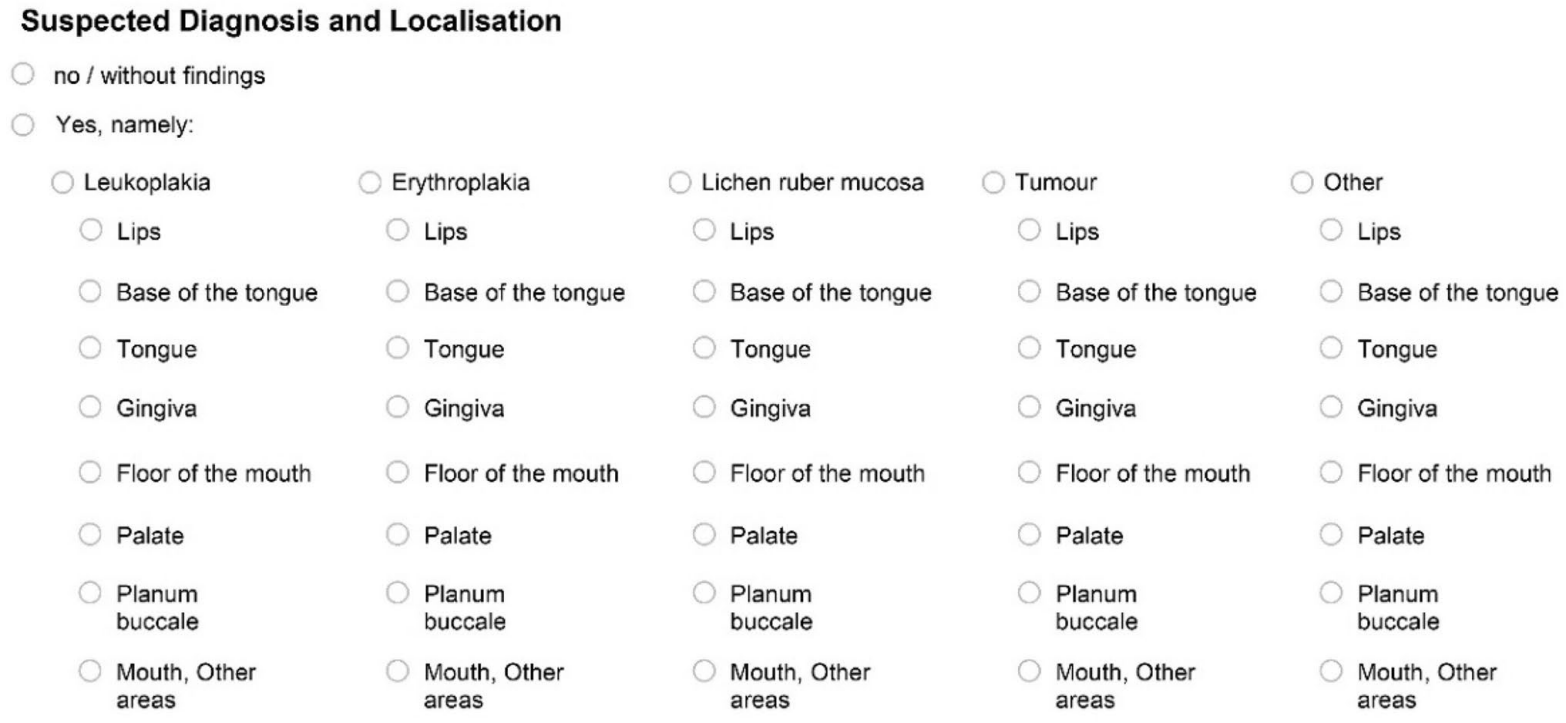
Presentation of the documentation sheet (text version)

**Fig. 2:**
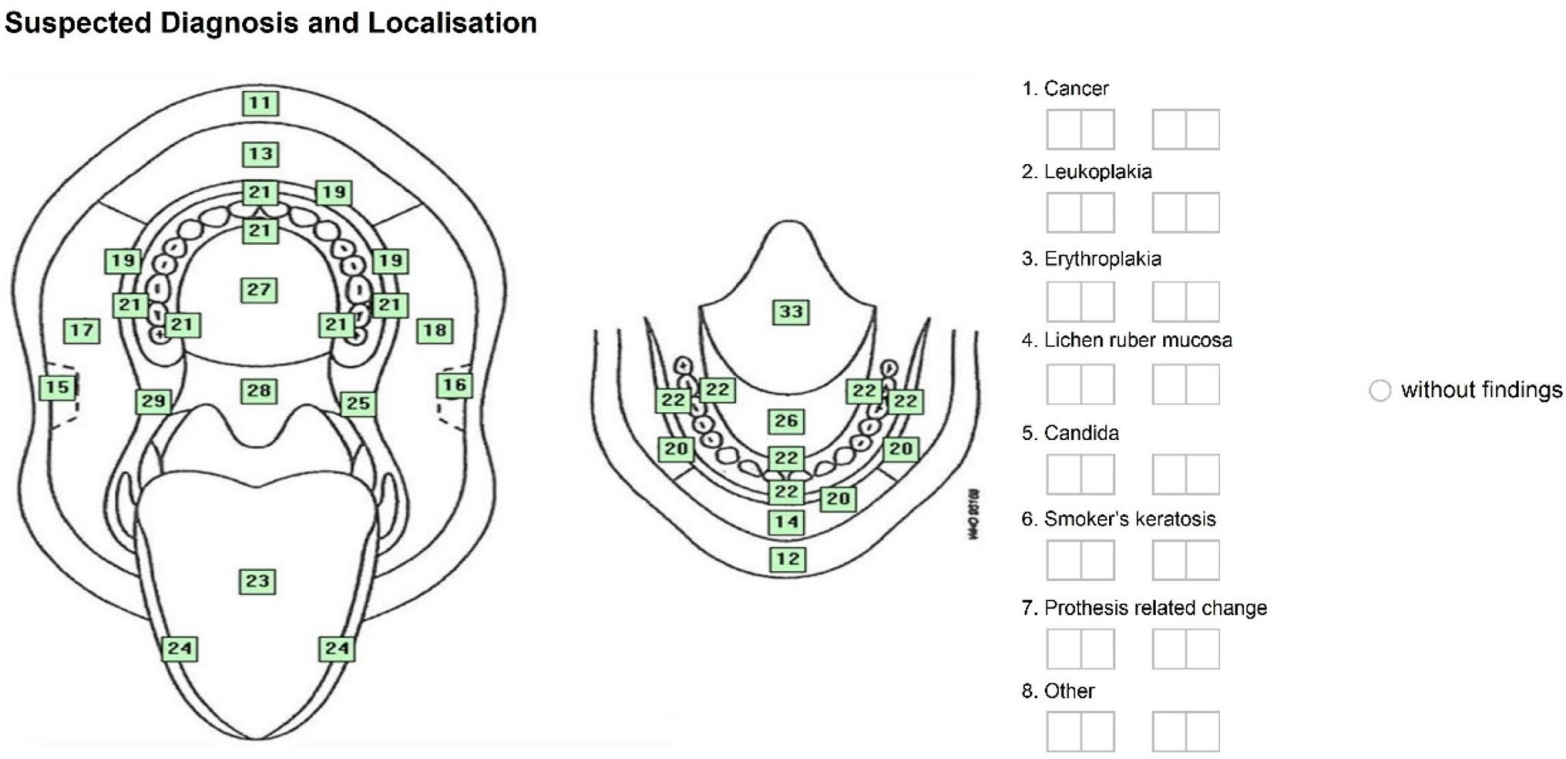
Presentation of the documentation sheet (graphic version)

Both versions of the documentation questionnaires had a matching ‘header’ section in which administrative patient data and questions about tobacco and alcohol use were to be recorded. They also featured the same questions on further treatment/referral and histological confirmation of the findings at the bottom of the sheets. The two versions differed in the number of suspected diagnoses and possible localization options (Figs. 1, 2). Each participating dentist received 100 sheets of both versions. These results belong to Phase I 'modelling' and will be elaborated in another manuscript (in preparation). Information on the sex of patients was not noted on the documentation sheet. However, using a reference list of common first names available from the Institute of Cancer Epidemiology e.V. at the University of Lübeck, the gender of most study participants was able to be deduced from their stated first name (Schwarz 2022).

Dentists were also asked to document when patients did not wish to participate in the study. This non-participation was recorded via a sheet including only the patient’s sex and year of birth. Four possible reasons for refusal and a free-text field were additionally provided.

### Procedure

Dentists were asked to record the oral mucosal inspection of all patients who came for a statutory dental check-up during a fixed period of 6 months and to use the documentation sheets provided. The aim was to document 200 patients per dentist during this period. This required informed consent from each eligible patient. The appropriate number of documents required was sent by mail to participating dentists with a detailed written explanation of the study procedure. Participating patient consents, non-participation forms, and oral mucosal documentation forms were continuously sent by return envelope from the dentists to the project management during this period. Two members of the working group (MJ, SW) were available to dentists for any questions or problems during the study period.

### Data processing and statistical analyses

Data from the paper-based documentation sheets for visual investigation of the oral cavity (case report form, CRF) were stored electronically in a data password-protected Access database. Personal data such as names and addresses of the study participants were kept separately from clinical/study data (i.e. identification code of the recruiting physician and of the private practice, date of the current check-up, sex and age of the patient, health insurance provider (statutory or private), alcohol and tobacco consumption, and findings of the check-up). After completion of the data collection, data cleansing was conducted via automated plausibility checks and manual checks of implausible data entries. If necessary, data entries in the Access database were corrected. Finally, we transferred the data into an SPSS file (pseudonymised dataset for analyses). Following the end of the recruiting period, we entered the information on study-decliners manually into a separate SPSS file. This information included the identification code of the recruiting physician and the private practice, year of the current check-up, the reason for declining, and sex and age of the patient. Information on the 25 participating dentists including the identification code of the recruiting physician and of the private practice, age, sex, years of working experience, type of practice, and workload and was entered manually into another SPSS file. Only MJ and AW had access to the database and SPSS files.

Statistical analyses involved descriptive statistics, using counts, percentages, means, and standard deviations. Tests for subgroup differences/association were conducted using the Welch test for numeric data and Chi-squared test for categorical data. Following an exploratory analysis approach we conducted a logistic regression analysis with the presence of a suspicious lesion as the dependent variable and age (in years), sex (male [reference], female, missing), alcohol (never [reference], < 1 per month, 2–4 times per month, 2–3 times per week, 4 or more times a week, missing information on alcohol consumption) and tobacco consumption (non-smoker [reference], current smoker, former smoker, missing information) as well as the reason for attending (regular check-up, suspect of lesion, missing) as potential predictors. A *p* value < 0.05 was considered statistically significant. All analyses were conducted using SPSS version 22.

## Results

### Description of participating dentists and recruitment process

**Fig. 3:**
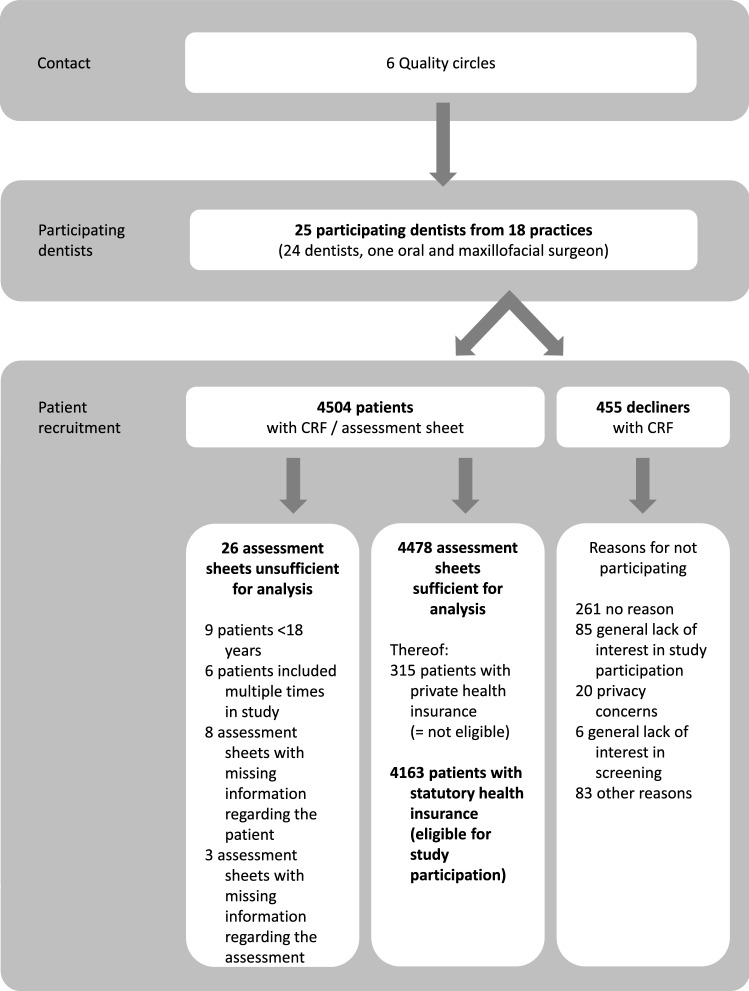
Flow Chart of the recruitment process

Out of a total of 53 participants, 25 dentists, one of them an oral and maxillofacial surgeon (response rate 47%), working in 18 different private practices located in Schleswig–Holstein (northern Germany) were recruited via quality circles and personal contacts of the principal investigator (KH) (Fig. 3). Table 1 displays the dentists’ characteristics. Two of the dentists had further qualifications as specialist dentists (in oral surgery and orthodontics respectively). Six dentists stated their main focus of activity via a specialist association: electroacupuncture according to Voll (*n* = 1), endodontics (*n* = 1), implantology (*n* = 3), periodontics (*n* = 1), prosthetics (*n* = 1), acupuncture (*n* = 1), and hypnosis (*n* = 1). One participant had a supplementary master’s degree in communication research.

**Table 1:** Characteristics of dentists

	Total population of dentists (*n* = 25)
Gender	
Female	6 (24%)
Male	19 (76%)
Age (years, mean, SD)	50.0 (SD: 20.0)
Working experience (years, mean, SD)	22 (SD: 10.0)
Experience as a dentist in private practice Working in own practise since (years, mean, SD)	19 (SD: 11.0)
Working in current practice since (years, mean, SD)	18 (SD: 11.0)
Type of practice	
Single person practice	9 (36%)
Working with one other dentist in practice	8 (32%)
Working with two other dentists in practice	8 (32%)
Working load	
Working days per week (mean, SD)	5 (SD: 1.0)
Working hours per week (mean, SD)	40 (SD:10.0)
Thereof hours (mean, SD) for:	
Treatment	33 (SD: 7.0)
Administration	7 (SD: 5.0)

In total, the dentists recruited about 4500 patient participants during the 6-month period of recruitment. Between 71 and 244 patients per dentist were successfully recruited, with the overall aim of 200 participating patients per dentist achieved by 11 dentists, and a further 4 dentists recruiting 198 or 199 patients.

### Description of participating patients

After exclusion of participating patients with incomplete document sheets (*n* = 11), of patients who did not meet the inclusion criteria (*n* = 18), and of patients with private health insurance (*n* = 315), a total of 4163 patients were eligible for analysis.

About 57.5% of participating patients were females, 41.0% were males, and 1.5% had missing information on sex (Table 2). Their mean age was 52 years. The majority of patients attended their dentists for a regular check-up, and less than 1% reported a suspected lesion as the reason for attending. About 58% of patients reported being non-smokers, and about 12% reported not consuming alcohol at all.

**Table 2:** Characteristics of participating patients and prevalence of suspicious lesions

	Total population (*n* = 4163)	Females (*n* = 2391)	Males (*n* = 1710)	*p*
Age (years, mean, SD)	51.9 (SD: 17.0)	51.9 (SD: 17.0)	52.0 (SD: 17.0)	(T = 0.144, df = 4501) 0.885^#^
Tobacco consumption				(Chi^2^ = 77.5, df = 3)
Non-smoker	2423 (58.2%)	1521 (63.6%)	856 (50.1%)	< 0.001^§^
Current smoker	914 (22.0%)	443 (18.5%)	458 (26.8%)	
Ex-smoker	731 (17.6%)	373 (15.6%)	355 (20.8%)	
Missing information	95 (2.3%)	54 (2.2%)	41 (2.4%)	
Alcohol consumption				(Chi^2^ = 218.4, df = 5) < 0.001^§^
Never	510 (12.3%)	356 (14.9%)	143 (8.4%)	
One a month	1277 (30.7%)	858 (35.9%)	399 (23.3%)	
2–4 times a month	1367 (32.8%)	763 (31.9%)	577 (33.7%)	
2–3 times a week	585 (14.1%)	249 (10.4%)	335 (19.6%)	
4 or more times a week	247 (5.9%)	81 (3.4%)	164 (9.6%)	
Missing information	177 (4.3%)	84 (3.5%)	92 (5.4%)	
Reason for attending				(Chi^2^ = 5.50, df = 2) 0.064^§^^,^*
Regular check-up	3931 (94.4%)	2274 (95.1%)	1599 (93.5%)	
Suspect of lesion	10 (0.2%)	3 (0.1%)	5 (0.3%)	
Missing information	222 (5.3%)	114 (4.8%)	106 (6.2%)	
Suspicious lesion	354 (8.5%)	176 (7.4%)	175 (10.2%)	*(*Chi^2^ = 10.1, df = 1) 0.001^§^

Data of 62 patients with unknown sex is not displayed and not included in the analysis of *p* values

*Cave: 2 cells have an expected number of cases of less than 5. Thus, interpret *p* value with care

^#^Welch-test

^§^Chi^2^-test

Females and males had the same mean age (M = 52 years; SD = 17) but differed regarding their reason for attending the dentist and their tobacco and alcohol consumption, with women being less frequently smokers or ex-smokers and drinking less alcohol compared to men (Table 2).

### Assessment sheets: suspected lesions

**Fig. 4:**
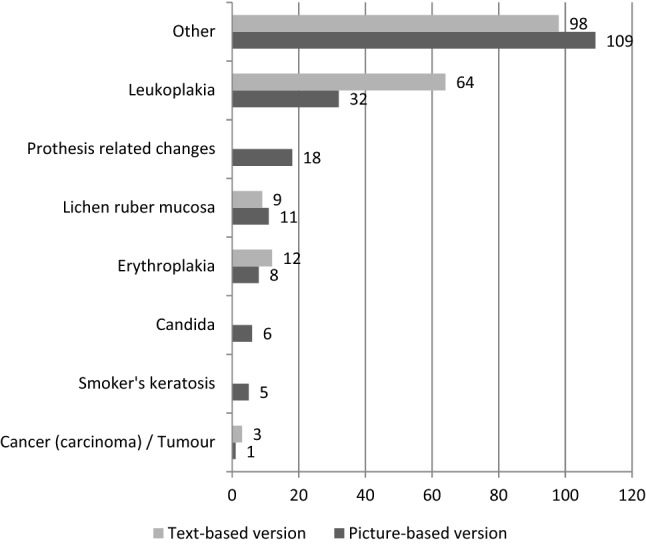
Frequency of suspected lesions according to the version of assessment sheet (*n* = 357 suspected lesions)

Overall, one or more suspected lesions were reported in 355 assessment sheets (8.5%). Men were more likely to show suspicious lesions than women (10 vs. 7%). Of all lesions, 49.9% were reported via the text-based version, slightly fewer than via the graphic-based version (50.1%; *p* = 0.647; χ^2^ = 0.210; df = 1; Table 3). Dentists differed regarding the amount of documented lesions (range 0.5–28% of all patients per dentist). The most common lesions were ‘other lesion’, followed by ‘leukoplakia’ (Fig. 4). ‘Prothesis-related changes’ were only reported via the graphic-based assessment sheet since these were not included in the text-based version.

**Table 3:** Suspected lesions as documented on assessments sheets according to version

Text-based version: suspected lesions as documented (*n* = 2124)		Absolute frequency	Relative frequency
Patients with any kind of suspected lesion		177	8.3%
Type of lesion	Localisation		
Leukoplakia		64	3.0%
	Lips	1	< 0.1%
	Base of the tongue	2	0.1%
	Tongue	10	0.5%
	Gingiva	15	0.7%
	Floor of the mouth	1	< 0.1%
	Palate	4	0.2%
	Planum buccale	20	0.9%
	Other area	16	0.8%
Erythroplakia		12	0.6%
	Lips	0	–
	Base of the tongue	1	< 0.1%
	Tongue	1	< 0.1%
	Gingiva	2	0.1%
	Floor of the mouth	2	0.1%
	Palate	6	0.3%
	Planum buccale	0	–
	Other area	1	< 0.1%
Lichen ruber mucosa		9	0.4%
	Lips	0	–
	Base of the tongue	0	–
	Tongue	0	–
	Gingiva	3	0.1%
	Floor of the mouth	1	< 0.1%
	Palate	0	–
	Planum buccale	5	0.2%
	Other area	1	< 0.1%
Tumour		3	0.1%
	Lips	0	–
	Base of the tongue	0	–
	Tongue	0	–
	Gingiva	0	–
	Floor of the mouth	0	–
	Palate	0	–
	Planum buccale	2	0.1%
	Other area	0	–
	Missing information on site	1	< 0.1%
Other		98	4.6%
	Lips	13	0.6%
	Base of the tongue	2	0.1%
	Tongue	18	0.8%
	Gingiva	10	0.5%
	Floor of the mouth	5	0.2%
	Palate	17	0.8%
	Planum buccale	24	1.1%
	Other area	11	0.5%
Picture-based version: suspected lesions as documented (*n* = 2039)			
Patients with any kind of suspected lesion		178	8.7%
Cancer (carcinoma)		1	< 0.1%
Leukoplakia		32	1.6%
Erythroplakia		8	0.4%
Lichen ruber mucosa		11	0.5%
Candida		6	0.3%
Smoker’s keratosis		5	0.2%
Prothesis related change		18	0.9%
Other		109	5.3%

Documented lesions were associated with sociodemographic patient characteristics such as age, sex, tobacco, and alcohol consumption as well as with the reason for attending in bivariate analysis (Table 4). In an exploratory regression analysis with age, sex, tobacco and alcohol consumption as well as the reason for attending as potential predictors, age (OR = 1.02), sex (OR_women_ = 0.79), and tobacco consumption (OR_current smoker_ = 1.73, OR_former smoker_ = 1.25), the reason for attending (OR_suspect_ = 15.1), but not alcohol consumption were significant predictors for the presence of a suspicious lesion. The regression model was statistically significant, χ^2^ = 85.173, *p* < 0.001, although the amount of explained variance was poor as shown by Nagelkerke’s R^2^ = 0.047.

**Table 4:** Characteristics of participating patients with and without suspicious lesions, results of exploratory multiple logistic regression model

	Suspicious lesion (*n* = 384)	No finding (*n* = 3766)	*p* (bivariate testing)	OR (95% CI)
Age (years, mean, SD)	51.4 (SD: 17.0)	52.0 (SD: 17.0)	(T = 6.155, df = 4112) < 0.001^#^	1.02 (1.02–1.03)
Sex			(Chi^2^ = 10.491, df = 2) 0.005^§^	
Males	174 (49.2%)	1536 (40.3%)		1
Females	176 (49.7%)	2215 (58.2%)		0.79 (0.63–0.99)
Unknown	4 (1.1%)	58 (1.5%)		0.44 (0.12–1.56)
Tobacco consumption			(Chi^2^ = 20.577, df = 3)	
Non-Smoker	174 (49.2%)	2249 (59.0%)	< 0.001^§^	1
Current Smoker	93 (26.3%)	821 (21.6%)		1.73 (1.31–2.29)
Ex-Smoker	70 (19.8%)	661 (17.4%)		1.25 (0.92–1.69)
Missing information	17 (4.8%)	78 (2.0%)		2.81 (1.43–5.49)
Alcohol consumption			(Chi^2^ = 11.987, df = 5)	
Never	38 (10.7%)	472 (12.4%)	0.035^§^	1
One a month	102 (28.8%)	1175 (30.8%)		1.16 (0.78–1.71)
2–4 times a month	107 (30.2%)	1260 (33.1%)		1.12 (0.76–1.66)
2–3 times a week	53 (15.0%)	532 (14.0%)		1.82 (0.75–1.85)
4 or more times a week	31 (8.8%)	216 (5.7%)		1.28 (0.76–2.16)
Missing information	23 (6.5%)	154 (4.0%)		1.07 (0.54–2.14)
Reason for attending			(Chi^2^ = 23.340, df = 2)	
Regular check-up	326 (92.1%)	3605 (94.6%)	< 0.001^§,^*	1
Suspect of lesion	5 (1.4%)	5 (0.1%)		15.1 (4.02–56.3)
Missing information	23 (6.5%)	199 (5.2%)		1.33 (0.81–2.20)

*Cave: 1 cell has an expected number of cases of less than 5. Thus, interpret *p* value with care

^#^Welch-test

^§^Chi^2^-test

### Assessment sheets: documentation quality

Slightly more text-based (51%, *n* = 2,124) than graphic-based assessment sheets (49%, *n* = 2,039) were used. The most common information missing was the identifying physician number for the dentist (43%) and the permanent establishment number (3%). The use of very heterogeneous formats for these numbers revealed problems with the standardized documentation of the data. With respect to patient identifying information, family names (*n* = 33), given names (*n* = 34), streets (*n* = 43), postal codes (*n* = 43), cities (*n* = 44 missing, *n* = 53 incomplete city names), and birth dates (*n* = 13) were less often incomplete or missing. Referral requests and further treatment were measured dichotomously (yes/no). The response option ‘no’ was rarely used, while ‘yes’ was used for 99 patients, thus the overwhelming majority of assessment sheets had missing information for this item. The same applied for the item regarding histopathological workup (‘yes’ was used for 18 patients). Plausibility checks during data entry led to automatic completion of the missing information for the latter two items. The date of examination was missing for 36 patients; information on whether written informed consent had been obtained was missing for 164 patients at first but was subsequently provided by the dentists on request.

Missing information for the text-based version was most commonly observed for the yes/no-item ‘without finding’. Furthermore, while the localization of a specific lesion was marked, the parent item indicating the kind of lesion was often not marked. Plausibility checks during data entry led to automatic completion of the missing information.

Missing information for the graphical-based version is most often related to the yes/no-item ‘assessment without finding’. Misinterpretation of the two boxes for identifying two different locations per lesion category as left and right was frequent (e.g. carcinoma: 22 and 22, instead of 22 and 23). In the case of more than two locations for one kind of lesion category, handwritten additions were made. In some rare cases, the category ‘other’ was used to indicate two different locations with (other) lesions, e.g. 17 and 18, while ‘no finding’ was reported for the yes/no-item ‘assessment without finding’. Plausibility checks during data entry led to automatic answer correction to ‘with finding’.

### Non-participation among patients

Although all dentists were asked to document basic data for non-participants on a case report form for decliners (‘decliner CRF’), two dentists did not provide any decliner CRFs. The other dentists provided either decliner CRFs (*n* = 455 CRFs; min. = 3, max. = 64), or reported the number of participants not invited for study participation (i.e. a further 455 patients with missing decliner CRFs). Reasons for not inviting these 455 patients were ‘time constraints’ (*n* = 68; 15%), ‘illness of dentist’ (*n* = 10; 2%), ‘other reasons’ (*n* = 3), or unknown reasons (*n* = 374; 83%).

Among the 455 non-participants with decliner CRFs, about 52.0% were females, 42% were males, and 6% had missing information on sex. Their mean age was 56 years (SD 19.4, range 16–93). The most common reasons for non-participation were ‘general lack of interest in study participation’ (*n* = 85, 19%) and ‘privacy concerns’ (*n* = 20, 4%), with missing information for more than half (*n* = 261, 57%) of non-participants.

## Discussion

The inspection of the oral mucosa for the detection of malignant lesions and tumours is simple, non-invasive, inexpensive, and safe. Furthermore, the oral cavity is easily accessible and thus the investigation should be routinely performed by practicing dentists. This was already explained by Reichart et al (1991) in a detailed guide for dentists as support for diagnostics (Reichart et al. 1991). In health care systems with a low threshold for dentist access, in particular, these screening initiatives should be attended regularly.

Dentists in private practice should therefore be involved in the design of studies aimed at drawing conclusions about the effectiveness of this screening. However, this requires study design to be integrated into the dental workflow and to take account of the reimbursement or billing system of the respective healthcare system, while at the same time meeting the required scientific standards for interventional study (Warnakulasuriya et al. 2015). For this reason, the study conducted combined clinical-epidemiological with methodological aspects regarding a feasibility study.

### Clinical-epidemiological aspects

Participating dentists documented various prevalent lesions in 8.5% of the patients within the 6-month duration of the study. These diagnoses referred not only to possible malignant lesions, but also included oral precursor lesions, lichen ruber mucosa, prosthesis-related changes, and candida. With regard to the risk factors of tobacco and alcohol consumption, we found that abnormalities were documented less frequently in women than in men (women 7%; men 10%). In light of previous research on the influence of smoking and alcohol consumption on the development of oral cancer (WCRF, AICR 2007), this is unsurprising, particularly because nicotine and alcohol consumption was reported more frequently in men than in women by the practising dentists. We, therefore, recommend that these two synergistic risk factors should be evaluated in a standardized way in future studies on the early detection of oral tumours and be reflected in recommendations for action, especially for dentists in private practice.

To date, few studies describing oral mucosal documentation in the dental practice setting have been published. A similar study to ours from England also asked dentists in private practice to examine 200 patients for possible lesions. But in contrast to our study, patients were invited to practices specifically for this examination, whereas our patients were continuously recruited during routine check-ups or patient-requested examinations. The proportion of detected lesions in the English study (14.1%) was significantly higher than in our study (8.5%). This was also true for the number of suspicious malignant lesions (4 vs 2.3%) (Lim et al. 2003). In a large US study, practicing dentists detected suspicious malignant lesions within a period of about half a year. The prevalence of detected lesions in this study was also higher than in our study at 9.5% (Kerr et al. 2020; Psoter et al. 2019).

Warnakulasuriya et al. (2015) described European studies on documentation of oral mucosal lesions in different settings in a comprehensive systematic review (Warnakulasuriya et al. 2015): studies in which examinations were performed in the workplace (Downer et al. 1995; Feaver 1997; Nagao et al. 2005), studies in which subjects were invited and not investigated in the office setting (Field et al. 1995; Jullien et al. 1995a, b; Jullien et al. 1995a, b), or investigated in clinics (Dombi et al. 2001; Harris et al. 2004; Vacher et al. 1999). A meta-analysis was not undertaken due to heterogeneity. Comparing the results with our study is not appropriate because of the very different settings and study interventions.

An analysis of a biopsy database from Toronto, Canada, revealed that the majority of biopsies sent and diagnosed were from office-based dentists. This underscores practice-based dentists’ critical role in early detection and points to the importance of performing this investigation and documenting it (Abadeh et al. 2019). A biopsy requires inspection of the oral mucosa. The main difference between this study and ours is that in our study the documentation of possible oral mucosal lesions was integrated into the everyday practice routine.

Another methodological approach to generating epidemiological data on oral mucosal lesions is, for example, field studies where dentists perform an inspection of the oral mucosa in volunteers as part of an invitation system. In contrast to the very good data on tumours of the oral cavity provided by epidemiological clinical cancer registries, the data on oral mucosal lesions is insufficient. In a Japanese study from the 1990s, residents over 40 years of age in a small town (around 50,000 inhabitants) were examined in a standardized way and followed up for four years. The authors described the number of diagnoses of leukoplakia and lichen ruber mucosa, for example, as high. Because the data were reported as age-adjusted incidence rates per person-years, direct comparison with other studies reporting the prevalence of suspicious lesions is not appropriate.

In Germany, two large population-based studies have for several years been conducted. In the German Oral Health Study (DMS), representative data on oral mucosal lesions were collected at intervals of several years. The prevalence data collected in DMS V were somewhat lower than the prevalence data observed in our study. This was true for leukoplakia (0.8 to 1.9% difference depending on the age cohort), with a slight difference described for lichen ruber mucosa (0.3% DMS V vs 0.4% in our study) as well as for tumours (0.2% DMS V vs 0.1% in our study) (Hertrampf 2016a, 2016b, 2016c). Another comprehensive longitudinal population-based study, the ‘Study of Health in Pomerania’ (SHIP), showed a slightly higher value of 0.9% for potentially malignant lesions (Kindler et al. 2021).

These different trends regarding the frequencies of detected lesions in studies in private dental practice and in population-based studies support the recommendation to conduct studies with an appropriate level of evidence on the effectiveness of screening in private practice. The aforementioned systematic review by Warnakulasuriya et al. (2015) also concluded that it is necessary to demonstrate the improvement in survival rates in the context of a study with an appropriate level of evidence to demonstrate the benefit of such measures. However, the working group acknowledged that conducting such an intervention study in a European setting would be challenging (Warnakulasuriya et al. 2015).

### Methodological aspects

We were able to demonstrate the feasibility in the principle of early detection of potential oral malignant lesions by conventional oral examination. Nevertheless, with regard to the methodological aspects, we were also able to generate a large number of indications for feasibility. In the following, we will elaborate on individual points we were able to identify as pitfalls and which may be resolved using control mechanisms.

It was not possible for the working group to determine whether every patient who was potentially suitable for the study really was consistently approached for participation during the documentation period. This aspect should be taken into consideration when drawing up the guidelines for recruitment of practices and possible sample size calculation for a large-scale follow-up study.

Our uncertainty here is supported by the dentists’ reports on patient inclusion in the study. Analysis of these reports suggests that dentists did not always recruit to the study patients who came for their six-monthly check-up. There were days or even weeks in almost every dental practice in which no patients were included in the study. This is also supported by the fact that the number of documentation sheets submitted by the dentists varied greatly (71 to 244 documentation sheets). Some reached the target of 200 documented patients within a short documentation period, while others did not reach the target at all within the specified documentation period of 6 months.

Regarding patient consents, there was a small proportion where informed consent was missing. Once the practices had been contacted by telephone to check whether written informed consent was available, dentists seemed to pay more attention to the requirement when filling out the form.

With regard to the documentation forms, problems arose with recording the submitting practices, as the ‘physician number’ required for identification had not been included on the sheet. This was a project start-up error, resulting in the forms subsequently having to be marked up by hand. With regard to a possible intervention study, these two essential aspects will need to be revised (the consent process and identification by the ‘physician number’), since the described procedure (telephone enquiry) would not be feasible in a large-scale study.

Of course, screening was only carried out for those people who actually attended the dentists for their check-up. Typically, these people are more health-conscious than those who do not visit the dentist regularly (healthy screenee effect). The effect on mortality may thus be smaller than under optimal conditions, in which population-based screening is performed with 100% participation.

### Strengths and weaknesses

The strength of the study is that it involved continuously recruited patients, i.e. a population-based cohort. Implementing the study via recruitment of dentists in private practice was facilitated by the well-established network enjoyed by the working group. During the study, continuous efforts at maintaining close contact with the dentists were required to be able to document compliance with the study protocol. The study followed the internationally required standards of the Medical Research Council (feasibility/piloting) for the development of intervention studies and thus provides a very good basis for the design of a corresponding follow-up study. Furthermore, the clinical data collected on patients confirmed that the results were in line with a systematic review and further international studies, even though prevalence was somewhat lower in this study.

The study also has weaknesses, however. Selection bias must be taken into account regarding dentist participation. It may be assumed that the dentists who agreed to participate were those who are interested and motivated. All of them received information about the study itself and the provided study material for the documentation of suspicious lesions but the dentists were not calibrated for the study and inter-rater reliability was not determined. Thus, it might be possible that—given the same clinical condition—one dentist would have neglected the presence of a suspicious lesion, while another dentist would have diagnosed a suspicious lesion. However, we assume the examiner bias to be low as the inspection of the oral cavity is part of the professional life of dentists and we assume that it is conducted with high diagnostic quality. Nevertheless, independent of this study, regular training on this topic would be recommended to improve diagnostic quality. This could reduce possible false-positive findings. The rationale for not-calibrating the dentists is that the working group is planning a large, population-based intervention study based on the present feasibility study. In a large-scale trial with more than 1000 dentists included a calibration will not be feasible. Furthermore, in our present study we wanted to reflect on the usual practice and quality of care. Furthermore, interpretation of the lesions detected must take into account that they were not verified, i.e. histologically confirmed, by either the dentists or by other institutions. Verification was not possible within the scope of this study. However, this verification must be given in a possible comprehensive interventional study. Since dentists already document digitally, the possibility of a digital documentation of the oral mucosa in the existing practice software with the necessary interfaces should be considered.

Compared with other countries, biopsies are performed by dentists in Germany only to a very limited extent. Individuals with suspicious lesions are sent to specialists or clinics (oral surgeons, maxillofacial surgeons), where the biopsy is then taken. And finally, for the interpretation of our exploratory logistic regression model one has to consider that the”suspicious lesion” rather than the histologically confirmed diagnosis of a malignancy served as the dependent variable and that the regression model itself was statistically significant, but the amount of explained variance was poor.

## Conclusion

The project demonstrated that a study on the early detection of oral and pharyngeal cancer can be carried out successfully in collaboration with dentists in private practice. Collection of clinical-epidemiological data on the early detection of oral mucosal lesions under everyday conditions proved both feasible and successful. It is therefore recommended that studies providing an appropriate level of evidence be conducted with dentists in private practice to enable conclusions to be drawn about the effectiveness of visual inspection of the oral mucosa as an early detection measure. This is also important since dentists in private practice consider one of their roles to be the early detection of malignant lesions via routine inspection of the oral mucosa and subsequent referral of patients to appropriate further diagnosis and therapy.

## Data Availability

Not applicable.
